# Media Consumption and Creation in Attitudes Toward and Knowledge of Inflammatory Bowel Disease: Web-Based Survey

**DOI:** 10.2196/jmir.7624

**Published:** 2017-12-08

**Authors:** Jacob Groshek, Miles Basil, Ling Guo, Sarah Parker Ward, Francis A Farraye, Jason Reich

**Affiliations:** ^1^ Department of Emerging Media Studies College of Communication Boston University Boston, MA United States; ^2^ Section of Gastroenterology Boston Medical Center Boston University Boston, MA United States

**Keywords:** inflammatory bowel disease, social media, health communication, social stigma

## Abstract

**Background:**

Inflammatory bowel disease (IBD) is a chronic gastrointestinal condition affecting over 5 million people globally and 1.6 million in the United States but currently lacks a precisely determined cause or cure. The range of symptoms IBD patients experience are often debilitating, and the societal stigmas associated with some such symptoms can further degrade their quality of life. Better understanding the nature of this public reproach then is a critical component for improving awareness campaigns and, ultimately, the experiences of IBD patients.

**Objective:**

The objective of this study was to explore and assess the public’s awareness and knowledge of IBD, as well as what relationship, if any, exists between the social stigma surrounding IBD, knowledge of the disease, and various media usage, including social media.

**Methods:**

Utilizing a Web-based opt-in platform, we surveyed a nationally representative sample (n=1200) with demographics mirroring those of the US Census figures across baseline parameters. Using constructed indices based on factor analysis, we were able to build reliable measures of personal characteristics, media behaviors, and perceptions and knowledge of IBD.

**Results:**

Among the American public, IBD is the most stigmatized of seven diseases, including genital herpes and human immunodeficiency virus/acquired immunodeficiency syndrome (HIV/AIDS). Additionally, IBD knowledge is generally low with 11.08% (133/1200) of the sample indicating no familiarity with the disease and 85.50% (1026/1200) of participants inaccurately answering two-thirds of the IBD index questions with which their knowledge was assessed. Increased knowledge of IBD is associated with lower levels of stigma. However, social media use is currently related to lower levels of IBD knowledge (*P*<.05). Furthermore, findings indicate that participants who most frequently engaged in producing social media content are less knowledgeable about IBD (*P*<.10), highlighting the potential for a dangerous cycle should they be contributing to a Web-based IBD dialogue.

**Conclusions:**

Greater efforts must be taken to stymie IBD misinformation across all media, but especially in social media channels, to increase IBD knowledge and reduce stigma surrounding IBD. These findings pave the way for further research qualitatively examining the pervasiveness of specific IBD messages found in today’s social media landscape and their impact on enacted stigmas so as to better equip providers and patient advocacy organizations with impactful communication solutions.

## Introduction

### The Social Stigma Challenge for Inflammatory Bowel Disease Patients

Inflammatory bowel disease (IBD) is a complicated and often debilitating disease with a variety of effects on the quality of life of patients. There are currently 5 million IBD patients worldwide. The symptoms of Crohn’s disease (CD) and ulcerative colitis (UC)—both of which are categorized under IBD—are often felt by sufferers to be embarrassing and disabling, and indeed the majority of patients with IBD report perceived stigma [1]. Perceived stigma is defined as the degree to which an individual comprehends a social stereotype to be against them [1]. Other forms of health-related stigma include enacted stigma (actual societal discrimination toward an individual) and internalized stigma (the extent to which the stigmatized individual actually believes in the social stereotype) [2]. Although embarrassment associated with IBD symptoms can, in some cases, lead to isolation, fear of being in public, and ostracization for patients, the stigma experienced by IBD patients has not been studied extensively. Given that, this study examines enacted stigma as it relates to IBD knowledge and different forms of media use on a nationally representative basis.

### Medical Conditions and Media Coverage—An Overview of Inflammatory Bowel Disease

Over the past decade, numerous health care and patient-related organizations have amplified the effort to increase awareness and reduce stigma of this chronic disease. World IBD Day is celebrated each year on May 19 in 36 countries to raise awareness of this disease, and the Crohn’s and Colitis Foundation (CCF) supports a range of informational events such as runs/walks, fashion shows, and education days. The CCF also maintains an active media presence on the Web in the form of interactive tutorials, webcasts, and social media to educate patients and clinicians on the recognition, diagnosis, and management of IBD.

At the same time, there is an increased use of social networking sites (SNS) for news and information among the general public. A Pew report found that social media platforms, namely, Facebook but also Twitter, now represent principal media sources for many individuals across age cohorts in the United States [3]. This study, therefore, builds on previous research on technology and health communication [4] by beginning to examine attitudes held by the general American public, how those attitudes relate to IBD knowledge, and specifically looks at different forms of Web-based media use as explanatory factors.

Although previous studies have explored IBD content and users on Twitter [5,6], there are no known studies that have explicitly examined the consumption of social media as well as the creation and sharing of content by users in these Web-based platforms with regard to how they contribute to IBD attitudes and knowledge. Similar studies on other medical conditions, such as antimicrobial resistance, have found that awareness is not only the product of exposure to certain media but is also related to personal experience and discussion [7,8].

We, therefore, incorporate these aspects within a theoretical framework of information exchange and social ties that are commonplace on the Web [9] to build a more well-developed communications perspective on a medical issue that is understudied in general but especially in terms of public attitudes and knowledge in an emerging media environment.

### Communicating IBD in a Public Health Framework and Emerging Media Environment

In the contemporary media environment, access to medical information is abundant and ubiquitous via the Internet and social media, and informational outlets that focus on diseases such as IBD have expanded to new arenas. Through mobile social media interfaces, it is possible for patients, doctors, researchers, friends, family, and even pharmaceutical companies to share information and foster discussion [10].

Within the medical community, the social media phenomenon represents an innovative and potentially transformative frontier in patient communication and the delivery of health information. The ways in which patients utilize social media, including YouTube, Facebook, and Twitter, among other platforms such as blogs and wikis, for assessing medical knowledge as it pertains to their own personal health are poorly understood and therefore of concern to medical and support personnel [5,11]. Some earlier work has suggested that Web-based communication may be especially effective in creating an egalitarian space to discuss and debate issues and to connect to others for the purpose of emotional support [12-14].

Indeed, although investigators such as Garrett [15] have argued that users posting inaccurate content may augment nonfactual information to spread widely and negatively shape attitudes, he nonetheless maintains that the Internet broadly acts as a mechanism to counteract and constrain the intentional efforts of users to propagate misinformation. Specifically, at an individual level, Garrett [15] points to how users may be deincentivized to publicly post information to social media that may not be accurate precisely because they are afraid of being proven wrong or accused of lying by other users who are experts in the area. In medical areas other than IBD, there are a number of cases where the Internet and social media have shown a positive relation to increasing public health awareness such as food recalls and related health risks [16,17]. In these cases, the public’s role in spreading news of outbreaks has been essential to the success of such information-driven campaigns.

However, other studies such as the work of Oh and colleagues [18] have found that rumors and misinformation can be exacerbated on social media, even in crisis situations or widespread health risks such as the Ebola or Zika virus. Similarly, findings from Grant and colleagues [19] indicate that interactive media can publicly undermine established medical knowledge by facilitating user-generated content that presents a compelling personal framework not rooted in fact or evidence. At this stage, there is a notable tension in the extant research where findings are not conclusive and may be specific to situation, media, or topic. As one example, a recent study by Groshek and colleagues [8] found that individuals who created and shared Web-based media were increasingly likely to be misinformed about antimicrobial resistance (AMR), as were more frequent consumers of traditional media, but that those consuming more Web-based information actually had a decreased likelihood of being misinformed about AMR. The only media factor that directly related to an increased likelihood of misusing antibiotics or other AMR-related products was consuming traditional media more frequently.

Considering this background as a whole, as well as taking into account the exploratory nature of this particular work on a disease that has no known cause or cure, and the way it is being introduced and received by the general public, this study thus proposes the following research questions (RQs):

- RQ1: How aware is the general public of IBD and what are the features of the social stigma surrounding IBD?
- RQ2: How knowledgeable is the general public regarding IBD and how does that knowledge vary across social groups?
- RQ3: How do different forms of media consumption and creation relate to the knowledge of IBD?
- RQ4: What relationship exists between the social stigma surrounding IBD, knowledge of the disease, and various media uses?

## Methods

This study included a sample of 1200 opt-in panel respondents through the Web-based survey company, Qualtrics. The demographics of this sample follow those of the US Census figures across baseline parameters. Specifically, 51.00% (612/1200) of respondents were female and the median age was 47 years with a mean of 46.12 (standard deviation, SD=16.70). By age cohorts, 11.75% (141/1200) were aged between 18 and 24 years, 36.25% (435/1200) between 25 and 44 years, 35.00% (420/1200) between 45 and 64 years, and 17.00% (204/1200) were 65 years and older, and in terms of ethnic groups, 74.08% (889/1200) of all respondents were white, 8.92% (107/1200) were African American, 9.25% (111/1200) were Hispanic or Latino, 5.58% (67/1200) were Asian, and a combined 2.17% (26/1200) indicated being either Native American, Pacific Islanders, or of other ethnic background.

Again, in maintaining a relatively close match to the general figures from the census, in terms of household income, there were 26.16% (313/1200) of respondents that earned US $24,999 or less, 28.67% (345/1200) made between US $25,000 and $49,999, 31.83% (382/1200) were in the US $50,000 to $99,999 income group, and 13.33% (160/1200) reported earning US $100,000 or more. Formal education levels showed that there were just 1.50% (18/1200) of respondents who did not graduate from high school or equivalent, 22.25% (267/1200) earned a high school diploma or General Educational Development (GED), there were 26.75% (321/1200) of respondents with some college education completed, 11.25% (135/1200) held a 2-year college degree, 28.08% (337/1200) graduated from a 4-year college, and 10.16% (122/1200) had earned some type of graduate degree such as an MBA, PhD, or JD, which also generally approximated the distributions of all Americans from the last census.

This study also constructed indices based on factor analyses to build reliable measures of concepts. For this block, all items were based on 1 to 10 scales of *very low* to *very high* responses that measured respondents’ feelings of social isolation (three items; Cronbach alpha=.881), overall life satisfaction (three items; Cronbach alpha=.770), and general trust in others (two items; Cronbach alpha=.817). There were another two statements that measured fatalism, which is a construct of the belief that individuals lack control to determine their future and that fate dictates one’s life (Cronbach alpha=.758). All of these indices were from a series of already validated instruments for each construct [20,21] and assist in developing parameters of control for subsequent analysis.

### Media Consumption and Creation

For this block of variables, respondents answered a series of questions about their media use. All media items measured the frequency of use on an ordinal 1 to 10 scale that had a range from *never* to *all the time*. Television and radio consumption were operationalized by five items that showed a high reliability (Cronbach alpha=.736), and two additional measures were combined about reading local and national newspapers (Cronbach alpha=.650) to round out traditional media consumption. Four additional items regarding the frequency of consuming social media content on the most frequently used channels in the dataset, namely, Facebook, Twitter, and YouTube, and streaming television such as Netflix that loaded strongly and reliably were also included (Cronbach alpha=.776). Finally, for this block, there were another four separate items that measured the frequency respondents reported creating and sharing Web-based media more actively by posting comments, uploading videos, or contributing to discussion threads on social network sites and adding material to wikis (Cronbach alpha=.922).

### IBD Perceptions and Knowledge

The only index constructed for this block was the compilation of 12 true/false items about IBD causes, symptoms, and possible cures, which can be found in Multimedia Appendix 1. These items were developed by a qualified team of subject matter experts, including IBD clinicians and researchers at the Boston University Medical Center and comprised statements that ranged in relative difficulty regarding the depth of knowledge required to answer correctly. For each item, respondents were awarded one point for a correct response and received a score of zero for each incorrect response, which built a theoretical range of IBD knowledge from 0 (no IBD knowledge) to 12 (full IBD knowledge). The executed Checklist for Reporting Results of Internet E-Surveys (CHERRIES) for this index is presented in Multimedia Appendix 2.

In addition, respondents were asked to self-assess their own familiarity with IBD on a scale of 1 (not at all familiar) to 10 (extremely familiar) and if they had been diagnosed with IBD themselves or if they knew anyone with the diagnosis. The final two measures in this block had respondents rank the degree of social stigma attached to IBD relative to seven other medical conditions and then to similarly rank nine symptoms of IBD from least to most embarrassing [22]. These are discussed in greater detail in answering RQ1 in the following section.

## Results

### IBD Perceptions and Knowledge

With respect to the general public’s awareness and perceptions of IBD as investigated in RQ1, respondents reported relatively low levels of familiarity with IBD on the whole, with a self-reported average of just 5.54 (SD 2.70) on a scale of 1 (not at all familiar) to 10 (extremely familiar). The mode for this item was 7, with 18.33% (219/1200) of the respondents indicating this familiarity level, which was followed by 4, which was indicated by 15.83% (189/1200) of the respondents, and then 1 (or no familiarity) being reported by 11.08% (133/1200) of the respondents. Similarly, just 9.67% (116/1200) of the respondents in this sample reported personally knowing someone who has been diagnosed with IBD, and only 3.17% (38/1200) indicated having been diagnosed with IBD themselves.

**Figure 1:**
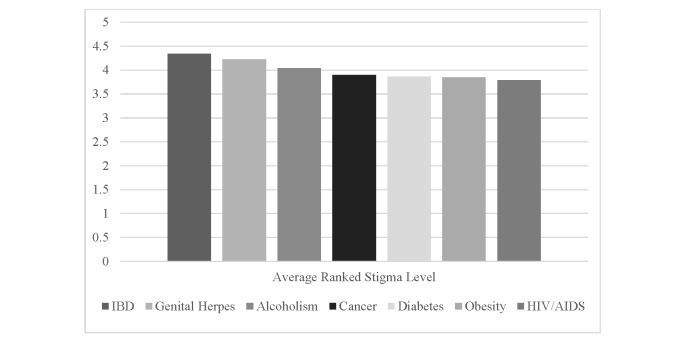
Average rankings of social stigma by disease (higher values indicate more stigma). N=1200 for all diseases.

The perception of IBD among the general public was highly negative, with respondents ranking IBD as having the greatest social stigma, on average, among seven health conditions considered here. When ranked on a scale of 1 (least social stigma) to 7 (most social stigma), IBD had the highest mean and was significantly more stigmatized in pairwise *t* tests than all the other conditions except genital herpes. As shown in Figure 1, in descending order from most to least social stigma, IBD (mean 4.34, SD 2.01) was followed by genital herpes (mean 4.22, SD 1.83; *t*_1,199_=1.37, *P*=.19), alcoholism (mean 4.04, SD 1.76; *t*_1,199_=3.44, *P*<.001), breast or testicular cancer (mean 3.90, SD 2.04; *t*_1,199_=6.04, *P*<.001), diabetes (mean 3.87, SD 1.95; *t*_1,199_=6.47, *P*<.001), obesity (mean 3.85, SD 1.82; *t*_1,199_=5.55, *P*<.001), and human immunodeficiency virus/acquired immunodeficiency syndrome (HIV/AIDS) (mean 3.79, SD 2.44; *t*_1,199_=4.80, *P*<.001).

There were no significant differences in terms of the level of stigma attached to IBD by men (mean 4.40, SD 1.87) or women (mean 4.28, SD 2.13) or across age groups, though social stigma for IBD was lowest among those in the age group of 18 to 24 years (mean 4.26, SD 2.29) and increased with each age cohort to a high among those 65 years and older (mean 4.50, SD 1.83). There were also no statistically significant differences across ethnic groups and income or education levels. Here it seems that respondents were somewhat uniform in their average rankings of IBD in social stigma relative to other conditions posed here.

The most embarrassing sequela of IBD of nine items considered here on a scale of 1 (least embarrassing) to 9 (most embarrassing) was presence of a stoma (mean 5.66, SD 2.81), which was followed by bloody diarrhea (mean 5.14, SD 2.57). Other externally noticeable symptoms such as skin sores (mean 5.10, SD 2.31), excessive weight gain (mean 5.08, SD 2.31), body odor (mean 4.95, SD 2.58), and the immediate and constant need to find a bathroom because of fecal urgency (mean 4.91, SD 2.44) also generated significant embarrassment. The remaining items of sudden dizzy spells, acne, and gas were within a mean of 4.74 to 4.71 in terms of their average rankings of embarrassment.

These findings suggest that IBD awareness among the general public is relatively low, yet it is highly stigmatized evenly across demographic factors, with both fecal-related and otherwise visible conditions ranking as most embarrassing and likely contributing to the stigma.

Moving forward to RQ2, which was concerned with IBD knowledge—as opposed to awareness—held by the general public, the average number of correct answers in this sample was 6.58 (or 54.8%; SD 1.77) items answered correctly when presented with 12 true/false items about IBD causes, symptoms, and possible cures. The minimum number of correct answers was 2 and the maximum number of correct answers was 11, indicating that not one respondent either answered all 12 items incorrectly or correctly. Altogether, this finding indicates that actual knowledge of IBD is quite low among the general public, with 85.83% (1029/1200) of this representative sample of respondents answering at least two-thirds of these items incorrectly.

As with perceptions of IBD, there was no statistically significant variation in IBD knowledge by gender, with averages of 6.66 (SD 1.82) for females and 6.49 (SD 1.71) for males. There were also no statistically significant differences by age groups, though it may be worth noting that the highest knowledge levels were observed among those 65 years and older (mean 6.69, SD 1.69), followed by those aged between 18 and 24 years (mean 6.69, SD 1.82). Differences in IBD knowledge also were not statistically significant by income levels, though those with the highest income were most knowledgeable (mean 6.70, SD 1.81), with corresponding declines in those averages to a low of 6.41 (SD 1.732) among the lowest income bracket.

Still, there were statistically significant differences across ethnic groups in terms of IBD knowledge, where respondents who indicated being either Native American, Pacific Islander, or of other ethnic background were most knowledgeable (mean 7.00, SD 1.74), on average, followed by white respondents (mean 6.71, SD 1.73), Hispanics or Latinos (mean 6.29, SD 1.86), Asian (mean 6.22, SD 2.04), and then African American respondents (mean 5.92, SD 1.55). Respondents reporting higher levels of formal education also demonstrated higher levels of IBD knowledge, and this was statistically significant. Specifically, those with 2-year, 4-year, or advanced graduate degrees had averages from 6.81 (SD 1.76) to 6.70 (SD 1.88), whereas those with some college education, a high school degree, or education less than finishing high school had means from 6.52 (SD 1.73) to 6.06 (SD 1.16).

### Media Consumption and Creation

Building off of the initial two RQs, RQ3 sought to understand how different forms of media consumption and creation relate to the knowledge of IBD. In this case, media use as it relates to the knowledge of IBD was examined using a hierarchical regression and found several statistically significant factors that helped to explain both increases and decreases in IBD knowledge. Here, in terms of media variables, two factors—creating Web-based content and relying on SNS—were statistically significant and both were negatively related to knowledge. More specifically, this model estimated that as creating Web-based content (B=−.06, standard error, SE=0.03, *P*<.10) and frequency of using social network sites for news and information (B=−.07, SE=0.03, *P*<.05) increased one unit, there was a related decline in IBD knowledge of .06 and .07 units, respectively.

Other factors that demonstrated a similar negative relationship were being older (B=−.13, SE=0.07, *P*<.10), more fatalistic (B=−.05, SE=0.02, *P*<.05), as well as considering IBD as more socially stigmatized (B=−.05, SE=0.03, *P*<.05). Apart from these items, there were several that related positively to IBD knowledge. These included being white (B=.32, SE=0.18, *P*<.10), being female (B=.20, SE=0.11, *P*<.10), being more educated (B=.13, SE=0.04, *P* ≤.001), and self-reporting a greater level of IBD familiarity (B=.04, SE=0.02, *P*<.10). This full model is summarized in Table 1.

The results of the regression were used to disaggregate the component items of statistically significant media factors. In a bivariate correlation matrix, each variable was separated from the creating Web-based content index and the using social network sites for news and information index. Here, it can be observed that IBD knowledge was most negatively related to uploading videos to platforms such as YouTube (*r*=−.19, *P*<.01) and more frequently contributing to wikis, similar, but not limited to Wikipedia (*r*=−.22, *P*<.01). Apart from those variables, the individualized measures of relying on different social networks, namely, YouTube (*r*=−.15, *P*<.01), streaming television such as Netflix (*r*=−.12, *P*<.01), and Twitter (*r*=−.12, *P*<.01) were the most negatively related to IBD knowledge.

Furthermore, the media variables themselves show mostly moderate but significant relationships to one another, indicating not only that the constructed indices are valid operationalizations but also that differences in the affordances of media platforms do connect negatively to the development of IBD knowledge among the general public. These results are summarized as a correlation matrix presented in Table 2.

Finally, when examining social stigma surrounding IBD, knowledge of the disease, and various media uses as proposed in RQ4, there were no significant correlations between media use and IBD stigma. The correlation between knowledge and attitudes was very weak (*r*=−.05, *P*<.10). These relationships were explored in greater detail by tracking the stigma assigned to IBD against ordinal rankings of IBD knowledge and each of the media use indices on 4-point scales from *very low* to *very high* that collapsed the variance in the original metrics based on even distributions. Here, none of the media variables again showed any relationship to IBD stigma whatsoever, but IBD knowledge was correlated.

Specifically, as knowledge of IBD increases along this ordinal scale, the stigma attached to it decreases (*F*_3,112.77_=2.30, *P*<.01, equal variances not assumed). Social stigma was highest (mean 4.93, SD 1.72) among respondents showing the least IBD knowledge, and the stigma level dropped at each increasing level of IBD knowledge to an average low of 4.02 (SD 1.77) for those respondents with the most IBD knowledge. Altogether, these findings suggest that the stigma surrounding IBD can best be diminished by increasing IBD knowledge through media campaigns that specifically target social media content and users.

**Table 1:** Regression model of inflammatory bowel disease (IBD) knowledge. N=1195, listwise deletion; overall adjusted *R*^2^=.071.

Variables		Coefficient (B)	Standard error (SE)	Beta (β)
**Step 1 (Demographics)**				
	Constant	6.066^a^	.970	
	Age in cohorts	−.128^b^	.071	−.065
	Being white	.322^b^	.182	.080
	Being African American	−.357	.235	−.057
	Being Asian	−.223	.268	−.029
	Being Native American/Pacific Islander/other	.542	.379	.044
	Gender (being female)	.200^b^	.110	.057
	Income	.074	.057	.042
	Education	.133^a^	.040	.104
	∆*R*^2^	.040^a^		
**Step 2 (Personal characteristics)**				
	Isolation	−.005	.024	−.007
	Life satisfaction	−.028	.028	−.035
	Trust in others	−.023	.028	−.026
	Fatalism	−.050^c^	.023	−.068
	∆*R*^2^	.019^a^		
**Step 3 (Media use)**				
	Television and radio	−.040	.035	−.046
	Newspaper	−.002	.025	−.003
	Creating Web-based content	−.057^b^	.033	−.072
	Social network sites	−.065^c^	.028	−.092
	∆*R*^2^	.020^a^		
**Step 4 (IBD^d^ background)**				
	Personally diagnosed with IBD	−.440	.305	−.044
	Know someone diagnosed with IBD	−.112	.186	−.019
	Self-reported familiarity with IBD	.036^b^	.021	.056
	Social stigma assigned to IBD	−.051^c^	.025	−.058
	∆*R*^2^	.007^c^		

^a^*P*<.001.

^b^*P*<.10.

^c^*P*<.05.

^d^IBD: inflammatory bowel disease.

**Table 2:** Bivariate Pearson correlation matrix for inflammatory bowel disease (IBD) knowledge and stigma in relation to regression-identified media variables. N=1200 for all cases.

Variables	1	2	3	4	5	6	7	8	9	10
1. IBD knowledge^a^	--^b^	−.05^c^	−.09^d^	−.19^d^	−.07^e^	−.22^d^	−.10^d^	−.12^d^	−.15^d^	−.12^d^
2. IBD stigma		--	−.01	−.01	−.01	−.03	−.01	−.04	−.02	−.01
3. Post comments			--	.52^d^	.63^d^	.42^d^	.49^d^	.42^d^	.41^d^	.30^d^
4. Upload videos				--	.60^d^	.70^d^	.36^d^	.47^d^	.47^d^	.43^d^
5. Create/share on SNS^f^					--	.44^d^	.51^d^	.46^d^	.40^d^	.33^d^
6. Write to wiki						--	.29^d^	.45^d^	.41^d^	.39^d^
7. Facebook news and information							--	.43^d^	.46^d^	.42^d^
8. Twitter news and information								--	.46^d^	.41^d^
9. YouTube news and information									--	.61^d^
10. Netflix/streaming TV news and information										--

^a^IBD: inflammatory bowel disease.

^b^--: perfect correlation with itself of 1.

^c^*P*<.10.

^d^*P*<.01.

^e^*P* ≤.05.

^f^SNS: social networking site.

## Discussion

This study sought to examine enacted stigma and public knowledge of IBD, which afflicts over 5 million people worldwide but does not have a long history of prominence on media, public, or policy agendas. As one of the first analyses of its kind on this topic, we carried out a generally representative nationwide survey of the American population to find that the general public is neither very aware nor very knowledgeable of IBD but does harbor high stigma for the disease. Simultaneously, although increased knowledge is inversely related to stigma, those who are most participatory in social media—through creating and consuming content—are found to be less knowledgeable on the topic. By shedding light on the underlying relationships between media participation, knowledge, and stigma, stakeholders will be more informed on the need to combat IBD misinformation and stigma with a multi-channel approach that not only encompasses but also pays particular attention to social media.

### Key Findings for Practitioners, Patients, and Advocacy Organizations

First, the findings identified that IBD awareness is relatively low among the general public in the United States. IBD is also simultaneously the most socially stigmatized medical diagnosis of seven other conditions considered in this study, which include genital herpes, alcoholism, and HIV/AIDS. Interestingly, the high level of IBD stigma was fairly evenly distributed over demographic and media use, and none of these characteristics were related to decreasing or increasing the social stigma attached to the disease. Although an explicit statistical link was not modeled here, it was apparent in survey results that fecal-related and other visible IBD symptoms such as stoma and skin sores ranked as the most embarrassing manifestations of IBD and presumably contribute to the pronounced social stigma that is attached to the disease across demographic, personal, and media factors.

Second, in addition to a high level of social stigma, IBD also suffers from a relatively low level of knowledge among the general public. When examining knowledge with 12 true/false items (Multimedia Appendix 1), the average number of correct answers was just over half, and a vast majority of all respondents answered at least two-thirds of all items incorrectly. Similar to IBD stigma, knowledge did not vary significantly across many demographic groups or personal characteristics, but it was shown that more educated respondents generally knew more about IBD, as did respondents claiming certain ethnic backgrounds, namely, those who are most typically affected [23].

Other factors were considered jointly and in greater detail in a hierarchical regression model, but it is worth noting here that in one-way analyses of variance (ANOVAs), knowledge of IBD declined significantly across increases in *all* forms of media use, which suggests that there is a systemic lack of accurate IBD information circulating offline and on the Web. In other words, there is not one mediated information channel that is related positively to IBD knowledge. In fact the opposite is true, which indicates that far greater efforts need to be made in raising IBD awareness through multiplatform media campaigns [24].

Each block of the regression model accounted for a statistically significant change in variance explained, but the most interesting findings suggest that increased consumption and creation on SNS are significantly and negatively related to IBD knowledge, even taking into account all other factors introduced to the model. Indeed, much like previous work on antimicrobial resistance [8], these findings suggest that creating Web-based content, and not just consuming news and information from certain sites or platforms, is part of a somewhat vicious cycle of misinformation [25]. Namely, the most prolific social media creators in this sample were also the respondents with the lowest levels of IBD knowledge. This finding requires us to again consider earlier works concluding that what high-volume producers share with others on social media is likely to affect those who regularly rely on social media for news and information, thereby perpetuating inaccurate beliefs [26-28], which, in this case, would contribute to misinformation about IBD, were it confirmed that they were producing content specific to that topic. Providers, then, must be aware that their patients may be receiving content that constitutes false information.

### Implications for Addressing and Alleviating IBD Stigma

Not unlike earlier studies on public Web-based information [15,29], the confluence of these findings suggests a linkage can be made from social media consumption and creation that relates negatively to IBD knowledge. This in turn is associated with increased IBD stigma, which is otherwise not directly influenced through various information channels. Specifically, and more importantly, increased IBD knowledge was the only factor to demonstrate a positive relationship to decreasing IBD stigma. Reducing this social stigma is, of course, a vital factor in raising acceptance and awareness of IBD in the public at large, and potentially mitigating some of the more serious quality of life issues faced by IBD patients such as work-related and other professional or personal interactions.

### Research Limitations and Opportunities for Further Exploration

One potential limitation that should be mentioned here is that although our sample was drawn from a pool of more than 32 million respondents to build a fully representative and generalizable sample, some limitations arguably remain for Web-based panels in this regard [30]. Even so, it is important to point out that similar stratified Web-based quota samples are regularly being engaged and employed for these sorts of analyses in the field of media research [31]. Another limitation is that the operationalization of social stigma was a nonspecific one, and thus does not explicitly provide details about what aspects of IBD are driving the high levels of stigma associated with the condition relative to other diseases. Likewise, we do not have knowledge measures on the other conditions that could form the basis of comparative work in future studies.

In addition, further research is warranted to more definitively investigate sources of IBD misinformation, including the qualitative nature of the content produced, as well as its life span and evolution in the emerging media environment, to better understand its effect on other social media consumers’ respective IBD knowledge. Such research would not only further clarify these findings but would also add critique to the divergent findings of existing health care communication literature on the role of social media in harming or harmonizing perceptions and knowledge. Most notably, such contribution may also inform stakeholders on specific social media tactics and messages useful in bolstering IBD knowledge and combating related stigmas.

Nonetheless, this study is perhaps the first to examine IBD in a communicative context, and likely one of only a few to map knowledge and public attitudes toward IBD in a broad sense. From the analyses outlined here, with a specific focus on the establishment of uniformly negative knowledge outcomes from all media, but social media consumption and creation in particular, this study advances a better understanding of media affordances [13] that can guide practitioners working in this area to build IBD knowledge and reduce its stigma.

In forging pathways toward that end, media activities promoting IBD causes, symptoms, and treatments should ideally take place across multiple levels, from initiatives facilitated through multinational organizations as well as direct social media interactions between physicians and patients. Perhaps most crucially, social media channels [24,25] must not be ignored because as demonstrated here, they are currently the most significant factors in shaping public knowledge of IBD, which in turn relates directly to stigma associated with the disease.

Considering these results, gastroenterologists and patient advocates must be aware of the impact social media has on negatively shaping public knowledge of IBD. At the moment, social media consumption and creation are both directly related to a lower level of IBD knowledge and thus indirectly related to increased IBD stigma, and this study represents a key platform for stakeholders to intervene positively in reshaping both IBD knowledge and stigma.

## Appendix

Multimedia Appendix 1 Index for IBD Knowledge with answers in bold.

Multimedia Appendix 2 Checklist for Reporting Results of Internet E-Surveys (CHERRIES).

## Abbreviations

AMR: antimicrobial resistance
ANOVA: analysis of variance
CCF: Crohn’s and Colitis Foundation
CD: Crohn’s disease
CHERRIES: Checklist for Reporting Results of Internet E-Surveys
IBD: inflammatory bowel disease
RQ: research questions
SD: standard deviation
SE: standard error
SNS: social networking sites
UC: ulcerative colitis
