# Supplementary material for: Media Consumption and Creation in Attitudes Toward and Knowledge of Inflammatory Bowel Disease: Web-Based Survey
Source: J Med Internet Res. 2017 Dec 8;19(12):e403. doi: 10.2196/jmir.7624 (PMC5741823; doi:10.2196/jmir.7624)
Supplement: Multimedia Appendix 1 [file jmir_v19i12e403_app1.pdf]

Multimedia Appendix

Appendix A
Index for IBD Knowledge

Instructions: Please answer the following questions to the best of your ability about inflammatory bowel disease.

**Index Statement**	**Response Options***	
Cultural norms, such as diet, are presumed to be a leading cause of IBD by medical professionals.	True	**False**
Lack of exercise is presumed to be a leading cause of IBD.	True	**False**
People of all ages are equally likely to be diagnosed with IBD.	True	**False**
Crohn's disease can affect any part of the gastrointestinal tract, from the mouth to the anus.	**True**	False
Crohn's disease and Ulcerative Colitis are the same disease.	True	**False**
IBS also known as irritable bowel syndrome is a type of inflammatory bowel disease.	True	**False**
Ulcerative Colitis and Crohn's disease tend to run in families.	**True**	False
Medications can cure inflammatory bowel disease.	True	**False**
Patients with IBD have an increased risk of developing colon cancer.	**True**	False
Most patients with Crohn's disease will require surgery in their lifetime.	**True**	False
Natural and homeopathic remedies have been shown to improve and/or cure IBD.	True	**False**
Most people with IBD will eventually need to stop working due to their disease.	True	**False**

*Correct answer selection made bold to assist reader comprehension.
