# Supplementary material for: Media Consumption and Creation in Attitudes Toward and Knowledge of Inflammatory Bowel Disease: Web-Based Survey
Source: J Med Internet Res. 2017 Dec 8;19(12):e403. doi: 10.2196/jmir.7624 (PMC5741823; doi:10.2196/jmir.7624)
Supplement: Multimedia Appendix 2 [file jmir_v19i12e403_app2.pdf]

Appendix B

Checklist for Reporting Results of Internet E-Surveys (CHERRIES)

**Item Category**	**Checklist Item**	**Explanation**
**Design**	Describe survey design	Target population: a nationally representative sample of the American public as per US Census figures.<br>Survey topics included feelings of isolation, attitudes toward fatalism, overall life satisfaction, social media use and participation, awareness of IBD including stigmas associations with IBD and IBD symptoms, and knowledge of 12 low-barrier facts about IBD. The IBD knowledge survey was designed by a team of subject matter experts including IBD clinicians and researchers from the Boston University Medical Center.
**IRB approval & informed consent process**	IRB approval	
	Informed consent	
	Data protection	No personal identifying information was collected.
**Development and pre-testing**	Development and testing	This study was prompted, in part, by earlier findings by the lead author regarding the dynamic role of social media in public awareness of antimicrobial resistance. Indexes investigating personal characteristics and media consumption were built upon a series of already validated instruments. The questionnaire regarding IBD knowledge was developed by a team of subject matter experts including IBD clinicians and researchers from the Boston University Medical Center.
**Recruitment process and description of the sample having access to the questionnaire**	Open survey versus closed survey	
	Contact mode	
	Advertising the survey	

**Survey administration**	Web/Email	
	Context	
	Mandatory/voluntary	
	Incentives	
	Time/Date	
	Randomizations of items or questionnaires	
	Adaptive questioning	
	Number of items	
	Number of screens	
	Completeness check	
	Review step	
**Response rates**	Unique site visitor	
	View rate (Ration unique site visitors/unique survey visitors)	
	Participation rate (Ratio unique survey page visitors/agreed to participation)	
	Completion rate (Ratio agreed to participate/finished survey)	
**Preventing multiple entries from the same individual**	Cookies used	
	IP check	
	Log file analysis	
**Analysis**	Handling of incomplete questionnaires	
	Questionnaires submitted with an atypical time stamp	
	Statistical correction	

Eysenbach G. (2004). Improving the quality of web surveys: the checklist for reporting results of interest e-surveys (cherries). *Journal of Medical Internet Research*, 6(3)e34 doi:10.2196/jmir.6.3.e34. http://www.jmir.org/2004/3/e34/
